# Artificial Intelligence–Based Chatbots for Promoting Health Behavioral Changes: Systematic Review

**DOI:** 10.2196/40789

**Published:** 2023-02-24

**Authors:** Abhishek Aggarwal, Cheuk Chi Tam, Dezhi Wu, Xiaoming Li, Shan Qiao

**Affiliations:** 1 Department of Health Promotion, Education and Behavior Arnold School of Public Health University of South Carolina Columbia, SC United States; 2 SC SmartState Center for Healthcare Quality (CHQ) University of South Carolina Columbia, SC United States; 3 UofSC Big Data Health Science Center (BDHSC) University of South Carolina Columbia, SC United States; 4 Department of Integrated Information Technology College of Engineering and Computing University of South Carolina Columbia, SC United States

**Keywords:** chatbot, artificial intelligence, AI, health behavior change, engagement, efficacy, intervention, feasibility, usability, acceptability, mobile phone

## Abstract

**Background:**

Artificial intelligence (AI)–based chatbots can offer personalized, engaging, and on-demand health promotion interventions.

**Objective:**

The aim of this systematic review was to evaluate the feasibility, efficacy, and intervention characteristics of AI chatbots for promoting health behavior change.

**Methods:**

A comprehensive search was conducted in 7 bibliographic databases (PubMed, IEEE Xplore, ACM Digital Library, PsycINFO, Web of Science, Embase, and JMIR publications) for empirical articles published from 1980 to 2022 that evaluated the feasibility or efficacy of AI chatbots for behavior change. The screening, extraction, and analysis of the identified articles were performed by following the PRISMA (Preferred Reporting Items for Systematic Reviews and Meta-Analyses) guidelines.

**Results:**

Of the 15 included studies, several demonstrated the high efficacy of AI chatbots in promoting healthy lifestyles (n=6, 40%), smoking cessation (n=4, 27%), treatment or medication adherence (n=2, 13%), and reduction in substance misuse (n=1, 7%). However, there were mixed results regarding feasibility, acceptability, and usability. Selected behavior change theories and expert consultation were used to develop the behavior change strategies of AI chatbots, including goal setting, monitoring, real-time reinforcement or feedback, and on-demand support. Real-time user-chatbot interaction data, such as user preferences and behavioral performance, were collected on the chatbot platform to identify ways of providing personalized services. The AI chatbots demonstrated potential for scalability by deployment through accessible devices and platforms (eg, smartphones and Facebook Messenger). The participants also reported that AI chatbots offered a nonjudgmental space for communicating sensitive information. However, the reported results need to be interpreted with caution because of the moderate to high risk of internal validity, insufficient description of AI techniques, and limitation for generalizability.

**Conclusions:**

AI chatbots have demonstrated the efficacy of health behavior change interventions among large and diverse populations; however, future studies need to adopt robust randomized control trials to establish definitive conclusions.

## Introduction

### Background

Artificial intelligence (AI)–driven chatbots (AI chatbots) are conversational agents that mimic human interaction through written, oral, and visual forms of communication with a user [1,2]. With the increased access to technological devices (eg, smartphones and computers) and the internet, AI chatbots offer the potential to provide accessible, autonomous, and engaging health-related information and services, which can be promising for technology-facilitated interventions. The existing digital therapeutic and telehealth interventions with didactic components, which enable health care providers to communicate with patients via digital platforms (eg, email and video call), have encountered several challenges, including relatively low adherence, unsustainability, and inflexibility [3,4]. AI chatbots offer the flexibility of on-demand support, personalized support and content, and consistent connectivity (sustainability), contributing to addressing the shortfalls of telehealth services. The overall *conversational flexibility* offered by AI chatbots in terms of communicating at anytime from anywhere offers a safe space to facilitate interactions with patients who feel or experience stigmatization while seeking health care services [5].

AI chatbots demonstrate their potential for effective behavior change through key steps of data processing in health-related conversations: data input, data analysis, and data output. First, AI chatbots can collect data sets from diverse sources: electronic health records, unstructured clinical notes, real-time physiological data points using additional sensors (eye-movement tracking, facial recognition, movement tracking, and heartbeat), and user interactions [5,6]. Second, the AI algorithm uses machine learning (ML) and natural language processing (NLP) techniques to identify clinically meaningful patterns and understand user needs [7]. Third, AI chatbots can mimic real-life human support by offering services that can assist users in achieving their health behavior goals [6]. Overall, by acknowledging user needs, demonstrating understanding, and delivering timely services tailored to user preferences (eg, goal setting, behavioral monitoring, and information or knowledge provision), AI chatbots have the potential to effectively deliver interventions that promote diverse health behaviors (eg, smoking cessation, physical activity, and medication adherence). AI chatbots can also be integrated into embodied functions (eg, virtual reality) that offer additional benefits, such as an immersive experience, which can catalyze the process of health behavior change [8].

### Prior Work

In the past decade, evidence regarding the feasibility and efficacy of AI chatbots in delivering health care services has focused on different health contexts and technological perspectives, and most of these chatbots aim to improve mental health outcomes. Of the extant systematic reviews on AI chatbots, 6 articles targeted at *assessing efficacy of AI-chatbots in enhancing mental health outcomes* [1,7,9-12], 2 examined the *feasibility* of AI-chatbots in health care settings [8,13], and 1 described the technical architectures and characteristics of the AI chatbots used in chronic conditions [14].

Given the merits of AI chatbots in health promotion, recent literature has paid increasing attention to the use of AI chatbots for health behavior changes. Oh et al [2] conducted a systematic review that assessed the efficacy of AI chatbots for *lifestyle modification* (eg, physical activity, diet, and weight management). However, the scope and inclusion criteria of this review had several limitations. First, this review did not distinguish between AI-driven chatbots and other chatbots. For example, the AI chatbots that performed *rule-based* or *constrained* conversation were included. Second, the selected studies targeted only a limited set of behaviors, including physical activity, diet, and weight management. Third, this review did not cover all platforms that could possibly deploy AI chatbots, the emerging technology platforms. For example, this review excluded the AI chatbots that were integrated into virtual reality, augmented reality, embodied agents, and therapeutic robots. Therefore, to provide a state-of-the-art understanding of AI chatbots for promoting health behavior changes, we were motivated to conduct a systematic review that covers the latest developments in AI chatbots, namely them being integrated into diverse devices (robots, smartphones, and computers) and diverse platforms (messenger and SMS text message), them performing “unconstrained” conversations, and them targeting a wide range of behavioral outcomes (smoking cessation, treatment or medication adherence, healthy lifestyle, and related health behavior domains). As such, this study aimed to provide critical evaluations of published empirical studies that describe AI chatbots’ intervention characteristics, components, or functionality and investigate their feasibility and efficacy in promoting a wide range of healthy behaviors on traditional and emerging platforms.

## Methods

### Data Sources and Search Algorithms

The study protocol of this systematic literature review followed the PRISMA (Preferred Reporting Items for Systematic Reviews and Meta-Analyses) guidelines [15] in each step. A comprehensive search was conducted in June 2022 by 3 authors (CCT, SQ, and AA) in 7 bibliographic databases, namely PubMed, IEEE Xplore, ACM Digital Library, PsycINFO, Web of Science, Embase, and JMIR publications.

The search was conducted using a combination of various keywords from 3 categories. The first category comprised keywords related to AI-based chatbot, including *chatbot*, *chatterbot*, *chatter robot*, *artificial intelligence*, *conversational AI*, *conversational agency*, *virtual agent*, *conversational agents*, and *bot*. The second category was related to health behaviors and included the keywords *health promotion*, *health behaviors*, *behavior change*, *substance use*, *alcohol use*, *drinking*, *cigarette use*, *smoking*, *drug abuse*, *drug use disorder*, *risk behaviors*, *lifestyle*, *exercise*, *nutrition behavior*, *sleep*, *adherence*, *body weight*, *physical activity*, *diet*, *risky behaviors*, *healthcare seeking behaviors*, *prescribed medical treatment*, *tobacco use*, and *vaping*. The third category focused on intervention study and included 1 keyword: *intervention*.

Keywords were organized using the following approaches: (1) keywords within one category were lined using the OR operator (eg, *chatbot* OR *conversational AI*), and (2) keywords across different categories were connected using the AND operator (eg, *chatbot* AND *health behaviors* AND *intervention*; Multimedia Appendix 1).

### Inclusion and Exclusion Criteria

This review selected empirical studies on health behavior interventions applying AI-based chatbot techniques according to the following inclusion criteria: (1) intervention research focusing on health behaviors; (2) empirical studies using chatbots; (3) chatbots developed upon existing AI platforms (eg, IBM Watson Assistant [IBM Corp]) or AI algorithms, such as ML, deep learning, natural language understanding, and NLP; (4) studies reporting qualitative or quantitative results on interventions; and (5) English articles published from 1980 to 2022 (as of June 2, 2022). Articles were excluded if they were (1) not full-text empirical studies (eg, conference abstracts or proposals); (2) intervention studies with chatbots based on non-AI methods, such as the rule-based approach; (3) studies that did not clarify their AI algorithms; or (4) studies that focused only on mental health and not on health behaviors.

**Figure 1:**
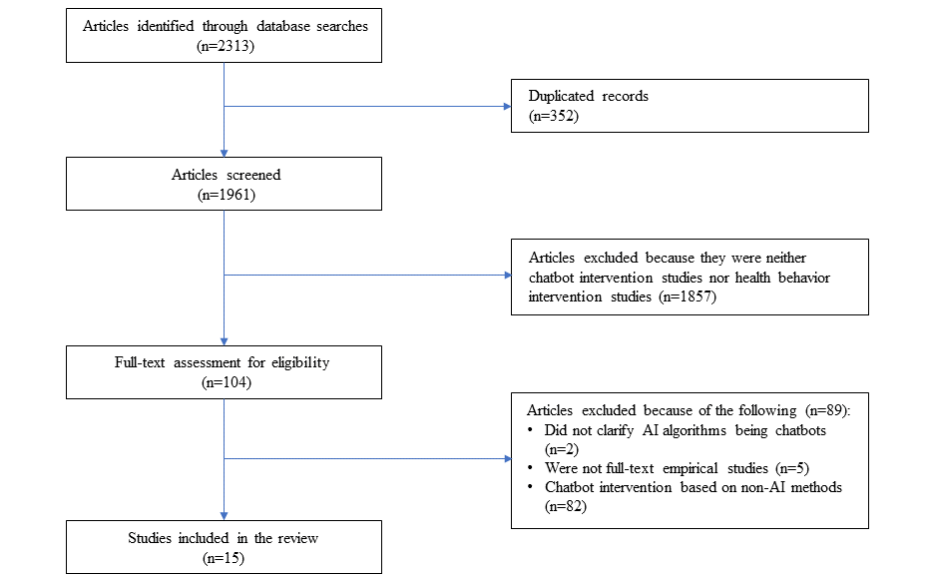
Eligibility screening process. AI: artificial intelligence.

A total of 1961 articles were initially retrieved and screened based on these criteria. Finally, 15 articles met the inclusion criteria and were selected for this review (Figure 1). Disagreements in selection were resolved through team discussions.

### Data Extraction and Quality Assessment

Several summary tables were used to extract information from the selected articles, including study characteristics (ie, author, publication year, study design, participants, age of the sample, sample size, country, and target health behaviors), chatbot-based intervention features (ie, chatbot types, chatbot components or functionality, settings, existing AI technology, input data sources, platform, theoretical foundation, and AI algorithms), and intervention outcomes (ie, health behavioral outcomes or primary outcomes, feasibility, usability, acceptability, and engagement).

Feasibility, acceptability, and usability did not have a consistent definition across the studies. Therefore, for the ease of comprehension and systematic representation, the authors categorized the data on feasibility, acceptability, and usability based on their definitions. Feasibility was defined as the *demand* of the intervention, that is, the actual use of the intervention and whether the intervention is *doable* in a certain setting [16]. For example, the number of messages exchanged with the chatbot and the engagement rate of the participants. Acceptability was defined as the quality of user experience with the AI chatbot [17], for example, the satisfaction score or number of likes to the interaction with the AI chatbot. Usability was defined as the level of contribution by the intervention to achieve the prespecified goals by users [18], such as the usability of the content provided by the AI chatbot in achieving health behavior goals.

Quality assessment of selected studies was performed in accordance with the National Institutes of Health’s quality assessment tool for controlled intervention studies [19]. This assessment tool suggests an evaluation of 6 types of bias risks. Specifically, (1) *the risk of reporting outcomes* based on ad hoc analyses was assessed based on prespecified outcomes; (2) *the risk of bias in the randomization process* was assessed based on randomized treatment allocation, concealment of allocation sequence (blinding), and similarity of groups at baselines; (3) *the risk of bias caused by deviations* from the intended interventions was assessed based on concealment of the assigned interventions from the participants, implementors, and evaluators; (4) *the risk of outcomes from unintended sources* was assessed based on measures to avoid the influence of other interventions and fidelity to the intervention protocol; (5) *the risk of bias in the measurement of the outcomes* was assessed based on concealment of assigned intervention from evaluators and validity and reliability of outcome measures; and (6) *the risk of bias in analysis* was assessed based on dropout rate, power calculation, and intent-to-treat analysis. Apart from the assessment of each risk type across studies, all studies were rated on the following scale: 1=compliant, 0=not clear, 0=not compliant, and 0=not reported or not applicable. A total score was calculated for each study.

AI techniques specific to AI chatbot interventions were also appraised using the CONSORT-AI (Consolidated Standards of Reporting Trials–Artificial Intelligence) extension guidance for AI studies [20]. We used a checklist of four domains: (1) whether the rationale for using AI was specified through the use of AI in the context of the clinical pathway, (2) whether the inclusion and exclusion criteria at the level of the input data and the description of the approaches to handle unavailable input data were specified, (3) whether the input data acquisition processes and the specifications of human-AI interaction in the collection of input data were described, and (4) whether the output of the AI algorithm and its significance in the context of the studies’ outcomes were described. The data extraction and quality assessment were conducted by 2 authors, CCT and AA, independently. All disagreements were resolved through sufficient discussions among CCT, AA, and SQ.

## Results

### Characteristics of the Reviewed Studies

The characteristics of the reviewed studies are summarized in Table 1. The included journal articles (N=15) were published in the following years: 2 (13%) from 2021, 3 (20%) from 2020, 6 (40%) from 2019, and 1 (7%) each from 2018, 2017, 2013, and 2011. Out of the 15 studies, 13 (87%) reported their geographical locations. All 13 studies were distributed across low-income countries, with 4 (31%) from the United States, 2 (15%) from Australia, and 1 (8%) from each remaining country (ie, South Korea, Spain, the United Kingdom, Japan, France, Switzerland, and the Netherlands). The sample size in the studies varied from 20 to 99,217, with a median of 116 and mean of approximately 7224 (SD 25,495.82) participants. Overall, 40% (6/15) of studies had >200 participants, followed by 27% (4/15) of studies with 100 to 200 participants, 13% (2/15) of studies with 50 to 100 participants, and 20% (3/15) of studies with <50 participants.

Out of the 14 studies that reported the mean age of the participants, most had adult participants aged 18 to 30 years (n=2, 14%), 30 to 40 years (n=3, 21%), 40 to 50 years (n=5, 36%), 50 to 60 years (n=1, 7%), and >60 years (n=1, 7%), with only 2 (14%) studies having participants aged <18 years. The selected studies included participants with diverse preexisting conditions: individuals with lower physical exercise and healthy diet levels (4/15, 27%), smokers (4/15, 27%), patients with obesity (2/15, 13%), patients with breast cancer (1/15, 7%), patients with substance use disorder (1/15, 7%), the general population (2/15, 13%), and Medicare recipients (1/15, 7%). The target health behaviors of the reviewed studies included promotion of a healthy lifestyle (physical exercise and diet; 5/15, 33%), smoking cessation (4/15, 27%), treatment or medication adherence (3/15, 20%), and reducing problematic substance use (1/15, 7%). Only 27% (4/15) of studies used randomized control trials (RCTs), and most of the studies (9/15, 60%) adopted a quasiexperimental design (ie, pre- and posttests) with no control group, followed by 7% (1/15) of studies with a cross-sectional design and 7% (1/15) of studies with a postexperimental research method.

**Table 1:** Characteristics of the reviewed studies (N=15).

Study	Study design	Participants	Average (SD) or median age (years)	Sample size	Country	Target health behaviors or purposes
Piao et al [21]	RCT^a^	Office workers	35	N=106 n=57 (intervention group) n=49 (control group)	South Korea	Healthy lifestyle (physical activity)
Maher et al [22]	Pre-post study^b^	Australians who did not meet Australia’s physical activity guidelines and not follow a Mediterranean dietary pattern	56.2 (SD 8)	N=31	Australia	Healthy lifestyle (physical activity and healthy diet)
Carrasco-Hernandez et al [23]	RCT	Smokers at an outpatient clinic	49.655	N=240 n=120 (intervention: chatbot + pharmaceutical treatment) n=120 (control: pharmaceutical treatment)	Spain	Smoking cessation
Stephens et al [6]	Pre-post study^b^	Youths with obesity symptoms at a children’s health care system	15.20	N=23	The United States	Treatment adherence (obesity)
Perski et al [24]	RCT	Smokers who purchased the Smoke Free app	N/A^c^	N=6111 n=1061 (intervention: chatbot + Smoke Free app) n=5050 (control: Smoke Free app)	The United Kingdom	Smoking cessation
Masaki et al [25]	Pre-post study^b^	Adult smokers with nicotine dependence	43.5 (SD 10.5)	N=55	Japan	Smoking cessation
Chaix et al [26]	Pre-post study^b^	Patients with breast cancer	48	N=958	France	Medication adherence
Calvaresi et al [27]	Pre-post study^b^	Smokers from Facebook communities	N/A	N=270	Switzerland	Smoking cessation
Galvão Gomes da Silva et al [5]	Qualitative study	Volunteers from School of Psychology’s pool	23	N=20	The United Kingdom	Healthy lifestyle (physical activity)
Stein and Brooks [28]	Pre-post study^b^	Adults with overweight and obesity (BMI ≥25)	46.9 (SD 1.89)	N=70	The United States	Healthy lifestyle (weight loss, healthy dietary, physical activity, and healthy sleep duration)
Crutzen et al [29]	Pre-post study^b^	Adolescents interested in the intervention	15	N=920	The Netherlands	Healthy lifestyle
Brar Prayaga et al [30]	Cross-sectional study (poststudy)	Medicare recipients	Median 71	N=99,217	The United States	Medication adherence
Prochaska et al [31]	Pre-post study^b^	American adults screened positive for substance misuse	36.8 (SD 10)	N=101	The United States	Reducing problematic substance use
To et al [32]	Quasiexperimental design without a control group	Individuals who were inactive (<20 min per day of moderate-to-vigorous physical activity)	49.1 (SD 9.3)	N=116	Australia	Healthy lifestyle (physical activity)
Bickmore et al [33]	RCT (4-arm)	Individuals in precontemplation or contemplation stages of change with respect to moderate-or-greater intensity physical activity or consumption of fruits and vegetables	33 (SD 12.6)	N=122	NR^d^	Healthy lifestyle (physical activity and healthy diet)

^a^RCT: randomized controlled trials.

^b^Pre-post studies had no control group.

^c^N/A: not applicable.

^d^NR: not reported.

### Intervention Study Quality Assessment

The results of the quality assessment are presented in Multimedia Appendix 2 [5,6,21-33]. *The risk of reporting outcomes* was low, as all the studies prespecified their outcomes and hypotheses. *The risk of bias in the randomization process* was low*.* All 27% (4/15) of RCTs adopted appropriate randomized treatment allocation and reported concealment of allocation sequence from the participants, and 75% (3/4) of them established similarity of groups at the baseline. The non-RCT studies (11/15, 73%) were not applicable for the assessment of the randomization process.

*Risk of bias of deviations from the intended interventions* was considered low to moderate. None of the included studies (N=15) reported concealment of the assigned interventions from the facilitators, evaluators, and participants, mainly because concealment from the persons providing and receiving behavioral, lifestyle, or surgical interventions is difficult [19]. *Risk of outcomes from unintended sources* was high. First, none of the studies reported any explicit measures to avoid the influence of other interventions on the outcomes or the existing intervention. In the case of RCTs (4/15, 27%), this bias was minimized because of the experimental setting of the interventions; however, for non-RCT studies (11/14, 73%), there was a high risk of bias owing to the potential effect of confounding variables. Second, most studies (13/15, 87%) did not report whether participants adhered to the intervention protocols.

*Risk of bias in the measurement of the outcomes* was moderate. First, none of the studies reported whether the assigned intervention was concealed from the evaluators. Second, 60% (9/15) of studies reported the reliability and validity of the outcome measures. In the remaining studies (6/15, 40%), the reliability and validity of the outcome measures were either not clear (3/6, 50%) or not reported (3/6, 50%). *Risk of bias in analysis* was moderate to high. First, studies with a ≥15% differential dropout rate between groups and ≥20% dropout rate for the intervention or control group were considered to have a high dropout rate [19]. Only 33% (5/15) of studies had a lower dropout rate than the cutoff limits, 20% (3/15) of studies did not report the dropout rate, and 47% (7/15) of studies had a higher dropout rate than the cutoff limits. Second, only 33% (5/15) of studies reported the use of power calculation to estimate a sample size that can detect a significant difference in the primary outcomes. Third, only 40% (6/15) of studies adopted an intent-to-treat analysis.

Among the 4 RCT studies, the study by Carrasco-Hernandez et al [23] reported the highest compliance relatively (8/12, 67%), followed closely by the study by Piao et al [21] (7/12, 58%). The study by Perski et al [24] reported compliance on only 50% (6/12) of the factors, closely followed by the study by Bickmore et al [33] (5/12, 42%). All 4 RCTs did not report *concealment of assigned intervention*, efforts to *avoid other intervention*, and *adherence* measures, and none of the RCTs were compliant with the dropout rates. Among the non-RCT studies, the studies by Maher et al [22] and Brar Prayaga et al [30] reported the highest compliance (5/9, 56%), followed closely by the studies by Masaki et al [25], Prochaska et al [31], and To et al [32] (4/9, 44%). Chaix et al [26] reported compliance on only 33% (3/9) of the factors, followed by the studies by Stein and Brooks [28] and Crutzen et al [29] (2/9, 22%). The remaining studies, that is, the studies by Stephens et al [6], Calvaresi et al [27], and Galvão Gomes da Silva et al [5], were complaint on only 11% (1/9) of the factors. Please note that the results of studies with <40% compliance need to be interpreted with caution.

### AI Quality Assessment

The AI component of the chatbots was evaluated to demonstrate AI’s impact on health outcomes (Multimedia Appendix 3 [5,6,21-33]). *Rationale for using AI* was prespecified in all the studies (N=15). *Characteristics and handling of the input data for AI* were described in only 7% (1/15) of studies. *Input data acquisition processes for AI* were mentioned in 87% (13/15) of studies. *Specifications of the human-AI interaction* were reported in the collection of input data in most of the studies (9/15, 60%). *The output of* *AI algorithms and its significance in context of the studies’ outcomes* were described in 87% (13/15) of studies. In conclusion, there was sufficient description for all factors, except for the input data characteristics and handling of unavailable input data.

### Outcomes of the Reviewed Studies

#### Efficacy

##### Quantitative Studies: Healthy Lifestyle

Out of the 15 studies, 7 (47%) studies [5,21,22,28,29,32,33] targeted healthy lifestyles, and 5 (33%) studies [21,22,28,32,33] assessed the efficacy of AI chatbots in promoting healthy lifestyles through (1) physical activity levels, (2) healthy diet, (3) blood pressure, and (4) BMI. First, 80% (4/5) of studies [33] reported an increase in physical activity. Stein and Brooks [28] reported that the increase in physical activity led to an average weight loss of 2.38% in 75.7% of the users (n=53). Maher et al [22] reported an increase in physical activity by 109.8 minutes (*P*=.005) and a decrease in the average weight and waist circumference by 1.3 kg (*P*=.01) and 2.1 cm (*P*=.003), respectively. Piao et al [21] reported significant between-group differences in the Self-Report Habit Index when controlled for intrinsic reward via chatbot enables app (*P*=.008). To et al [32] reported that the participants recorded more steps (*P*<.01) and more total physical activity (3.58 times higher; *P*<.001). Moreover, the participants were also more likely to meet the physical activity guidelines (95% CI 3.31-12.27) at follow-up. However, only Bickmore et al [33] reported no significant differences among the conditions in the International Physical Activity Questionnaire (*P*=.37).

Second, 20% (3/15) of studies [22,28,33] reported an improvement in diet. Stein and Brooks [28] reported that the percentage of healthy meals increased by 31% and the percentage of unhealthy meals decreased by 54%. Maher et al [22] reported an increase in the mean of Mediterranean diet (healthy meal) scores by 5.7 points (*P*<.001). Bickmore et al [33] reported that the group with only diet-related intervention consumed significantly more fruits and vegetables than the groups which received only physical activity intervention or both physical activity and diet intervention (*P*=.005); however, there were no significant differences among different groups for weight (*P*=.37). Third, Maher et al [22] assessed blood pressure level after intervention as a secondary outcome; however, the mean improvement in systolic blood pressure (-0.2 mmHg; *P*=.90) and diastolic blood pressure (−1.0 mmHg; *P*=.54) were not significant. Fourth, only To et al [32] reported that the decrease in BMI was not significant (95% CI −0.37 to 0.11). In conclusion, there were significant differences in the primary outcomes of interest (physical activity level and healthy diet) in all studies aimed at improving healthy lifestyles.

##### Quantitative Studies: Smoking Cessation

Out of the 15 studies, 4 (27%) studies [23-25,27] assessed the efficacy of AI chatbots in smoking cessation. Perski et al [24] reported that the intervention group had 2.44 times greater odds of abstinence at the 1-month follow-up than the control group (*P*<.001). Masaki et al [25] reported that the overall continuous abstinence rate results (76%, 12 weeks; 64%, 24 weeks; and 58%, 52 weeks) were better than the results of the outpatient clinic (calculated through the national survey) and the varenicline (medication for smoking cessation) phase 3 trial in the United States and Japan. Masaki et al [25] reported a decrease in social nicotine dependence (mean −6.7, SD 5.2), tobacco craving (mean −0.6, SD 1.5), and withdrawal symptoms (mean −6.4, SD 5.8), their secondary outcomes. Calvaresi et al [27] reported that 28.9% of the participants completed their smoking cessation goal 3 months after the last cigarette. This result was 10% higher than that of the previous edition of the smoking cessation program, which did not include chatbot support. Carrasco-Hernandez et al [23] reported that smoking abstinence (exhaled carbon monoxide and urine cotinine test) was 2.15 times (*P*=.02) higher in the intervention group than in the control group. However, none of the secondary clinical measures (health-related quality of life, healthy lifestyle, and physical activity) showed any differences between the groups. In conclusion, there was evidence indicating significant long-term and short-term effects of chatbot-based interventions on smoking cessation.

##### Quantitative Studies: Substance Misuse

Out of the 15 studies, only 1 (7%) study [31] aimed at reducing problematic substance use. Prochaska et al [31] reported a significant increase in the confidence to resist urges to use substances (mean score change +16.9, SD 21.4; *P*<.001) and a significant decrease in the following: substance use occasions (mean change −9.3, SD 14.1; *P*<.001) and the scores of Alcohol Use Disorders Identification Test-Concise (mean change −1.3, SD 2.6; *P*<.001), 10-item Drug Abuse Screening Test (mean change −1.2, SD 2.0; *P*<.001), Patient Health Questionnaire-8 item (mean change 2.1, SD 5.2; *P*=.005), Generalized Anxiety Disorder-7 (mean change 2.3, SD 4.7; *P*=.001), and cravings scale (68.6% vs 47.1% moderate to extreme; *P*=.01).

##### Quantitative Studies: Treatment or Medication Adherence

Out of the 15 studies, 3 (20%) studies [6,26,30] targeted medication or treatment adherence, but only 2 (67%) of these studies [26,30] reported the efficacy of AI chatbots in increasing treatment or medication adherence through timely and personalized reminders. Brar Prayaga et al [30] reported that out of the total refill reminders (n=273,356), 17.4% (n=47,552) resulted in actual refill requests. Furthermore, 54.81% (26,062/47,552) of those requests resulted in medications being actually refilled within 2 hours. Chaix et al [26] reported that the average medication adherence rate improved by more than 20% in 4 weeks (*P*=.40) through the prescription reminder feature. In conclusion, there was evidence indicating a significant increase in medication adherence rate through chatbot use; however, cultural differences were observed in chatbot use.

##### Qualitative Study: Healthy Lifestyle

Only one study conducted a qualitative analysis, that is, the study by Galvão Gomes da Silva et al [5]. This study reported that NAO, a social robot, enhanced immediate motivation toward activities such as meeting friends and families and increasing willpower through mindfulness techniques. The participants also reported that they felt more self-aware and were open to sharing their goals with others. However, the study reported mixed results regarding the achievement of physical activity goals.

#### Feasibility

The outcomes of the selected studies are reported in Multimedia Appendix 4 [5,6,21-33]. Out of the 15 studies, 11 (73%) reported the feasibility of AI chatbots in terms of (1) safety [22] (ie, no adverse events were reported), (2) messages exchanged with the chatbot [6,26,29,31,32], (3) retention rate [22,26], and (4) duration of engagement. Only 7% (1/15) of studies [22] reported the chatbot’s safety in terms of the absence of adverse events. Many studies reported the total number of messages exchanged with the chatbot (5/15, 33%) [6,26,29,31,32]; however, only 7% (1/15) of studies reported the exact proportion of user-initiated conversations (approximately 30%) [6], which depicted the participants’ level of interest in having health-related conversations with the chatbot. Few studies [22,23,26] reported variability in engagement and retention rate across study durations. Overall, 7% (1/15) of studies reported a gradual decrease in the retention rate (users who sent at least 1 message per month for over 8 months)—from 72% (second month) to 31% (eighth month) [26]. Similarly, another study reported that engagement was highest at the first month and reduced gradually, becoming lowest at the 12th month [23]. Similarly, another study [22] reported a decrease in check-ins by 20% midprogram, followed by an increase to 70% in the final week. Overall, there was strong evidence of decrease in engagement with the chatbot over time. It is important to note that there was inconsistency in terms of engagement metrics across different studies. Overall, there was very less evidence on the safety of chatbots and some evidence on the feasibility of chatbots in terms of the total and mean number of health-related messages exchanged; however, there are no defined thresholds to determine whether the number of messages exchanged demonstrates feasibility. It was also interesting to note that in one of the studies (7%), the engagement rate decreased over time but increased at the end [22].

#### Acceptability

Out of the 15 studies, 7 (47%) reported acceptability and engagement of AI chatbots in terms of (1) satisfaction and (2) provision of a nonjudgmental safe space. In the case of satisfaction, 7% (1/15) of studies reported that approximately one-quarter of the participants liked the messages [32], and another (7%) reported that the satisfaction of the participants with the web-based agent was above average [33]. In 7% (1/15) of studies, only one-third of the participants reported the desire to use the chatbot in the future [32], and in another study (7%), on average, the participants reported a below-average desire to continue with the agent in the future [33]. Similarly, another study (7%) reported that the participants liked the chatbot’s advice one-third of the times [25]. Only 7% (1/15) of studies [26] reported high user satisfaction (93.95%). Overall, the proportion of satisfied participants or the overall satisfaction rate in terms of content likeability and the future use of chatbots was less than 50%. Overall, 20% (3/15) of studies reported that the AI platforms offered a nonjudgmental safe space for users to share detailed and sensitive information [5,26,29]. The participants reported that the chatbots provided a personal space and time to think and respond uninterruptedly [5]; to share personal and intimate information such as sexuality, which they could not share with their physician directly [26]; and to ask questions regarding sex, drugs, and alcohol as they considered chatbots to be more anonymous and faster than information lines and search engines [29].

#### Usability

Out of the 15 studies, 11 (73%) reported the usability of AI chatbots in terms of (1) ease of using the chatbot, (2) outside-office support, (3) usability of the content, and (4) technical difficulties. Overall, the ease of using chatbots was low to moderate. The ease of use was dependent on the participants’ smartphone skills, platform’s user interface, and cultural sensitivity in the chatbot’s design. One study reported that chatbots were used to offer outside-office support to the participants, demonstrating the potential of AI chatbots to offer sustainable and continuous support [6]. Most of the studies (9/15, 60%) demonstrated the usability of the content shared by the chatbot through self-report measures and the number of times chatbot services were used. Generally, the content was considered reliable, concise, of high quality, and easy to understand. Some studies reported high scores, on average, for personalized messages and diverse information. Some studies reported the need to remove ambiguity in the content. Masaki et al [25] reported the number of *calls* made to the AI nurse to seek assistance for smoking impulses or side effects (mean 1.7 times, SD 2.4), demonstrating the need for AI chatbot at a critical time. Overall, the quality of the recommendations provided by AI chatbots can be further improved to make them more feasible for the participants to implement, along with improvements in the design of the user interface. In 7% (1/15) of studies [32], most participants reported technical issues in using the chatbot (82.3%), one of the reasons being that they stopped receiving the chatbot messages during the study period (84.1%). In conclusion, although the chatbots effectively offered outside-office support, the ease of using the chatbot and the usability of the content need to be further improved by providing credible and doable recommendations in a user-friendly design interface.

#### Chatbot Intervention Characteristics

##### Behavior Change Theories and Chatbot Functionality

The chatbot intervention characteristics are summarized in Multimedia Appendix 5 [5,6,21-33]. In more than half of the studies (9/15, 60%), the AI chatbots’ content, features, and interface were designed based on a theory. Each study critically selected theories based on the intervention goals and target beneficiaries. The cognitive behavioral therapy (CBT) was used in Tess [6], Lark Health Coach (HCAI) [28], and Woebot [31] to devise strategies that enhance self-efficacy and sustain behavior change. In Tess [6], CBT was clubbed with the theory of emotionally focused therapy and motivational interviewing to assist the behavioral counseling of adolescent patients. Similarly, in Woebot [31], CBT was clubbed with motivational interviewing and dialectical behavior therapy to provide emotional support and personalized psychoeducation to resist substance misuse. The theory of motivational interviewing was also used to devise interview questions addressed by NAO [5] (the social robot) and the motivation reinforcement messages provided by Bickmore et al’s [33] Chat1. In HCAI [28], CBT was clubbed with the Diabetes Prevention Program’s curriculum to develop content for conversations on weight loss.

The habit formation model, which explains the relationship among cues, behaviors, and rewards, was used to develop the reminder system in Healthy Lifestyle Coaching Chatbot (HLCC). The Mohr’s *Model of Supportive Accountability*, which states that the inclusion of human support in digital interventions increases engagement, was used to mimic human support in Smoke Free app (SFA) [24] to increase accountability and belongingness. Furthermore, SFA’s [24] behavior change techniques were coded against a 44-item taxonomy of behavior change techniques in individual behavioral support for smoking cessation. The transtheoretical model (TTM) of behavior change was used by Carrasco-Hernandez et al [23] to determine message frequency for the AI chatbot. Similarly, TTM was used in Bickmore et al’s [33] Chat1 to design the behavioral monitoring process, which included reviewing progress, identifying barriers, and solving problems. The Capability, Opportunity, Motivation, Behavior model, the core of the Behavior Change Wheel, a behavioral system focusing on 3 components—capability, opportunity, and motivation—was used in To et al’s [32] Ida to set goals, monitor behavior, reinforce behavior change through motivational messages. Social cognitive theory was also used in Ida [32] to facilitate therapeutic dialog actions (ie, talk therapy) and homework sessions outside the agent counseling sessions. Apart from the use of theories, expert consultation and institutional assistance were adopted to develop AI chatbots’ content. Mental health experts were consulted to develop and deliver customized messages through Tess [6], whereas 2 national health promotion institutions in the Netherlands developed the content for Bzz [29]. In conclusion, most studies either adopted a set of critically selected behavior change theories or consulted domain experts (individuals or institutions) to develop behavior change strategies.

On the basis of the behavior change theories, the AI chatbots had multiple functionalities that contributed to efficacious outcomes. First, 53% (8/15) of studies targeted *behavioral goal setting*. These chatbots targeted healthy lifestyles (7/8, 88%; HLCC, Paola [22], SFA [24], NAO [5], HCAI [28], Ida [32], and Chat1 [33]) and the reduction of substance misuse (1/8, 12%; Woebot [31]). The chatbots with goals related to healthy lifestyles enabled users to set physical activity and dietary goals with push alarms to maintain daily routines and monitor weight. Second, 73% (11/15) of studies used *behavioral monitoring*. The chatbots that targeted healthy lifestyles (5/11, 45%; HLCC, Paola [22], HCAI [28], Ida [32], and Chat1 [33]) enabled behavioral monitoring by consistently providing feedback through performance content and pictures, weekly check-ins, and data-based inputs on performance. The chatbots that targeted smoking cessation (3/11, 27%; DigiQuit [23], SFA [24], and SMAG [27]) offered data-driven feedback on health indicators through web-based diaries and graphs. The chatbots that targeted medication or treatment adherence (2/11, 18%; Vik [26] and mPulse [30]) offered timely reminders to take medications or refill medicines. The chatbot that targeted the reduction in substance misuse performed mood tracking and regular check-ins to maintain accountability (1/11, 9%; Woebot [31]).

Third, 53% (8/15) of studies offered *behavior-related information*. The chatbots that targeted healthy lifestyles (3/8, 38%) offered educational sessions on the benefits of physical activity (Ida [32]) and healthy diet (Paola [22]) and information on sex, drugs, and alcohol (Bzz [29]). The chatbots that targeted smoking cessation (4/8, 50%; DigiQuit [23]; SFA [24]; CureApp Smoking Cessation [CASC], [25]; and SMAG [27]) educated users on the benefits of being a nonsmoker, implications of abrupt cessation, and alternatives to smoking. The chatbot that targeted medication or treatment adherence (1/8, 12%; Vik [26]) offered information on the health issue (breast cancer) for which the users were taking medication.

Fourth, 53% (8/15) of studies reported *motivation reinforcement*. The chatbots that targeted healthy lifestyles (3/8, 38%) offered feedback on behaviors (HLCC and Ida [32]) and reinforced optimism to change behaviors through planning and imagining change (NAO [5] and Ida [32]). The chatbots that targeted smoking cessation (4/8, 50%) reinforced motivation through personalized messages based on TTM (DigiQuit [23]), scoreboards and trackers of milestones (SFA [24]), and motivational messages (CASC [25] and SMAG [27]). The chatbot that targeted reduction in substance misuse focused on motivation and engagement through individualized weekly reports to foster reflection (Woebot [31]).

Fifth, 27% (4/15) of studies provided *emotional support*. Of them, 3 studies (75%) targeted healthy lifestyles, and 25% (1/4) targeted reduction in substance misuse. Among the interventions that targeted healthy lifestyles, Tess [6] offered empathetic health counseling or compassionate care through ML-driven emotional algorithms; NAO [5], the social robot, expressed empathy through humanized robot interaction, and HCAI [28] mimicked health professionals’ empathetic health counseling. The intervention that targeted reduction in substance misuse, Woebot [31], offered empathic responses by tailoring to users stated mood.

Sixth, 7% (1/15) of studies (CASC [25]) delivered *provider-recommendation system* services. CASC [25] offered advice and counseling support to physicians. Seventh, 47% (7/15) of studies reported 24*7 availability of the AI chatbot. The chatbots that targeted healthy lifestyles (4/7, 57%; Paola [22], Tess [6], HCAI [28], and Bzz [29]) offered on-demand support, unlimited conversations, and answers to infinite number of questions. The chatbots that targeted smoking cessation (3/7, 43%) offered on-demand emergency support via an AI nurse (CASC [25]), support during periods of high cravings (SMAG [27]), and unlimited availability for conversations (SFA [24]). Eighth, 13% (2/15) of studies promoted activities beyond conversation with chatbots. Chat1 [33] offered homework assignments, whereas Woebot [31] required mindfulness exercises, gratitude journaling, or reflecting upon patterns and lessons already covered. In conclusion, AI chatbots offered personalized, real-time feedback and on-demand support to users continuously and indefinitely.

##### Infrastructure of the Chatbots: AI Techniques

Most of the studies (10/15, 67%) deployed different AI techniques to deliver personalized interventions: NLP, ML, hybrid techniques (ML and NLP), Hybrid Health Recommender System, face-tracking technology, and procedural and epistemological knowledge–based algorithm. ML-driven emotional algorithms were used in Tess [6] and HCAI [28] to provide empathetic counseling or compassionate care (emotion-based response). The AI algorithm analyzed users’ messages (voice or text based) to identify and categorize their emotions. Thereafter, the chatbots provided both emotional and strategic support to the users. NLP and ML techniques were used in Paola [22], Vik [26], Ida [32], and Woebot [31] to identify and categorize user intents and entities by analyzing unstructured messages. Bickmore et al’s [33] Chat1 used procedural and epistemological knowledge–based AI algorithms that facilitated therapeutic dialog actions (talk therapy). A hybrid technique combining NLP and conversational AI or ML was adopted by mPulse [30] to ensure smooth, continuous, and uninterrupted conversations. Hybrid Health Recommender System was adopted by Carrasco-Hernandez et al’s [23] AI chatbot to personalize messages based on user demographics, content (interest of the user), and utility (ratings on each message by the user). Face-tracking technology was integrated into NAO [5] (the social robot) to track participants’ faces to humanize the interaction experience. The remaining chatbot studies (5/15, 33%) specified the use of AI to personalize the chatbot interaction but did not elaborate on the AI techniques adopted. In conclusion, most studies targeted *personalized services* through different AI techniques.

##### Infrastructure of the Chatbots: Logistics

The chatbots used multimodal channels of communication with the users. All chatbots except NAO [5] (14/15, 93%) used text-based communication with the users, among which 2 (14%; Tess [6] and Vik [26]) chatbots also used voice-based communication. NAO [5] used only voice-based communication, as it was deployed via a social robot. The AI chatbot–based interventions were implemented for different durations: 0 to 2 months (3/15, 20%), 2- to 5 months (7/15, 47%), 5 to 9 months (2/15, 13%), 9 to 12 months (2/15, 13%), and >12 months (1/15, 7%). Out of the 13 chatbots that reported the frequency of engagement, all chatbots, except NAO [5], interacted with the users daily. NAO [5] interacted only once because it was delivered in person through a social robot. The AI chatbots were either integrated into existing platforms or delivered independently. Vik [26], SMAG [27], Tess [6], and Ida [32] were integrated into Facebook (Meta Platforms, Inc) Messenger. Tess [6] was also available on WhatsApp (Meta Platforms, Inc), Amazon Alexa (Amazon.com, Inc), Google Home (Google LLC), and mobile SMS. HLCC [21] was integrated with KakaoTalk (Kakao Corp), a popular messenger app in South Korea, and mPulse [30] was integrated with mobile SMS. The remaining chatbots (8/15, 53%) were delivered independently. The chatbots were deployed using different devices. All chatbots except NAO [5], Ida [32], and Chat1 [33] (12/15, 80%) were deployed through smartphones, among which 3 (27%; Vik [26], SMAG [27], and Bzz [29]) chatbots were also deployed through computers. Chat1 [33] was deployed only through computers. NAO [5] was deployed through a social robot, and Ida [32] was deployed through Fitbit Flex 1 (Fitbit LLC). Three chatbots (HLCC, Paola [22], and Ida [32]) integrated an existing AI-driven conversational platform, that is, the Watson conversation tool (HLCC and Paola [22]) and Dialogflow, an advanced Google ML algorithm (Ida [32]). In conclusion, AI chatbots can be deployed through accessible devices and platforms, indicating their potential for reaching remote and large populations.

##### Infrastructure of the Chatbots: Input Data for Personalized Services

To deliver personalized services using AI chatbots, most chatbots or studies (9/15, 60%) required input data on the users’ background, goals, and behavioral performance and chatbots’ usability and evidence-based content. The users’ background information or baseline characteristics were collected by 4 AI chatbots. Paola [22] measured the baseline level of physical activity and Mediterranean diet; SFA [24] measured time to first cigarette and cigarettes per day; CASC [25] measured demographics, motivation levels for smoking cessation, number of cigarettes smoked per day, and years of smoking; SMAG [27] measured demographics and type of smoking dependence; and Tess [6] used electronic health records. Information on the users’ goals, that is, who, when, where, what, and how, was collected by 3 chatbots. HLCC [21] asked the users (office workers) to set realistic stair climbing goals, Paola [22] enabled the users to set dietary goals and daily step target every week based on the previous week’s outcomes, and SFA [24] asked the users to set the target quit date for smoking. Real-time feedback on usability was collected by 3 chatbots. DigiQuit [23] collected feedback on the message content and timing, Tess [6] collected data on the usefulness of the message, and Vik [26] collected data on the relevance of the reminders. Real-time feedback on the behavioral performance of the users was collected by 5 chatbots. HLCC [21] collected performance content and pictures; Paola [22] collected data on daily steps and dietary patterns; Vik [26] collected data on medication adherence levels; SMAG [27] monitored the users’ smoking levels along with information on location, alone or accompanied, ongoing activity, and mood to create smoking profiles for them; and HCAI [28] gathered data automatically through sensors on phones and integrated devices such as wearables and self-reported information such as on dietary consumption. Overall, 20% (3/15) of studies used evidence-based content apart from the user data. Tess [6] used clinical scripts targeted at behavior change, CASC [25] used national guidelines on counseling support, and HCAI [28] used content from the Diabetes Prevention Program’s curriculum. In conclusion, most studies used diverse input data sets, indicating the need to collect comprehensive and essential input data for delivering personalized services.

## Discussion

### Principal Findings

#### Overview

The results of this review demonstrate the potential of AI chatbots to deliver efficacious, effective, and feasible health behavior interventions. However, the high risk of internal validity, lack of sufficient description of AI techniques, and lack of generalizability of the selected studies suggest the need for further research with robust methodologies to draw definitive conclusions. Regardless, the review identified practical and research implications of intervention strengths and limitations of the existing studies with potential future directions.

#### Primary Outcomes

This review found that AI chatbots were efficacious in promoting healthy lifestyles, including physical exercise and diet (6/15, 40%), smoking cessation (4/15, 27%), treatment or medication adherence (2/15, 13%), and reduction in substance misuse (1/15, 7%). These findings are consistent with previous systematic reviews that reported the use of AI chatbots for improvement in physical activity levels and improvement in medication adherence [2,5], treatment adherence [14], adherence to self-management practices [1], smoking cessation [12], and reduction in substance abuse [12].

#### Secondary Outcomes

The review found that AI chatbots reported mixed results in terms of feasibility, acceptability, and usability. In the case of feasibility, evidence on the safety of chatbots was quite less because only 7% (1/15) of studies reported safety [22]. As there were no predefined or standard thresholds on the number of message exchanges that demonstrates feasibility, it was difficult to interpret whether the AI chatbots were feasible. Some of the previous systematic reviews reported feasibility in the form of engagement with AI chatbots; however, the feasibility metrics differed across studies, and there was strong evidence regarding decrease in engagement rates over time [11,13,14]. Similarly, AI chatbots had mixed results in terms of acceptability. Although AI platforms offered a nonjudgmental safe space for the users to share detailed and sensitive information anonymously, the proportion of satisfied participants or the overall satisfaction rate of chatbots was less than 50%. These findings are partially aligned with previous systematic reviews that reported acceptability [11,13,14] and on-demand availability, accessibility, and satisfaction [7]. Similarly, the results on usability were mixed. Some studies reported that AI chatbots were efficient in offering outside-office support and high reliability and understandability of the content, whereas other studies reported a lack of personal connection with chatbots, poor smartphone skills among the participants, impractical recommendations by the chatbot, and technical challenges such as those where the participants stopped receiving the chatbot messages during the study period. These findings are partially aligned with previous systematic reviews on AI chatbots that reported that chatbots provided helpful information and were easy to use [7]. Overall, our mixed results regarding feasibility, acceptability, and usability are partially aligned with the findings of the existing systematic reviews that reported heterogeneity in these secondary outcome measures and results across studies [1,2,7,9-11].

### Implications of Intervention Characteristics

#### Theoretical Foundation for Behavior Change

The fundamental characteristics of the AI chatbots played a critical role in determining efficacious outcomes. First, the majority of the studies (9/15, 60%) used critically selected behavior change theories in the design and delivery of the AI chatbots. Our findings suggested that the integration of behavior change theories such as CBT, TTM, motivational interviewing, emotionally focused therapy, habit formation model, and Mohr’s Model of Supportive Accountability resulted in the delivery of consistent motivational support to users through goal setting, monitoring or tracking behaviors, and reinforcement. These strategies not only contributed toward better primary and secondary outcomes but also solved several challenges in the traditional face-to-face intervention models from users’ standpoint, such as limited connectivity with the expert, lack of consistent motivation, and lack of access to diverse information over time. Previous systematic reviews also reported that the use of CBT [2,11], habit formation model, emotionally focused therapy, and motivational interviewing [2] for designing behavior change strategies for AI chatbots contributed to better engagement, user motivation, and health behavior outcomes. More interdisciplinary collaboration between behavioral health experts and computer scientists is required to develop theory-based AI chatbots for behavior change interventions.

#### Free-Flow Conversation

Second, in all studies, *free-flow conversations* rather than *rule-based or constrained conversations* with AI chatbots enhanced user experience through the personalization of services, delivery of diverse information, and choice of user-initiated conversation. By contrast, rule-based chatbots offer limited user experience through constraints on the input data, a finite set of conversations that are task oriented and straightforward, and a lack of user-initiated conversations. This finding is consistent with previous systematic reviews that reported the need for greater personalization in AI chatbots through feedback on user performance, accountability, encouragement, and deep interest in the user’s situation [13]. Similarly, Milne-Ives et al [8] reported a need for greater interactivity or relational skills, empathetic conversations, and a sense of personal connection with the user through compassionate responses.

The need for greater interactivity can also be associated with the fluctuations in user engagement found in 13% (2/15) of studies [22,23]. In one of these studies, the engagement rates decreased gradually as the intervention progressed [23], and in the other study, the engagement rates decreased significantly by midprogram but increased to 70% in the final week, that is, the 12th week [22]. This is a novel finding that has not been reported in previous systematic reviews. As specified in the AI chatbot design literature, user engagement is dependent on the chatbot’s ability to understand the user’s background, build a relation, be persuasive, and offer quick feedback [34]. Therefore, it is critical for AI chatbots to establish appropriate rapport or relationship with the user through personalized and compassionate interactions for a sustained and engaging intervention. AI experts must establish pathways for comprehensive real-time data collection to produce accurate and personalized responses.

#### Nonjudgmental Space

Third, in 20% (3/15) of studies, the humanistic yet nonhumanistic construct of AI chatbots provided a safe space for the users to discuss, share, and ask for information on sensitive issues [5,22,23,35]. The ML-driven emotional algorithms offered the potential for perceiving and understanding human emotions [36], whereas the *nonhuman interaction* experience or the lack of interaction with a *real* human made it easier for the user to self-disclose sensitive information [37]. Thus, AI chatbots demonstrate their potential for intervening with vulnerable populations, especially in terms of stigmatized issues. For example, adolescence is characterized by high social anxiety; therefore, adolescents perceive stigma in seeking services on sensitive issues such as mental health disorders. In such scenarios, AI chatbots offer sufficient privacy and anonymity for adolescents to express their thoughts and emotions freely. This finding is consistent with a previous systematic review that reported the use of anonymity for encouraging users to freely express their emotions [13].

#### Scalability

Fourth, most studies (8/15, 53%) reported that the AI chatbots have a low threshold for integration into existing services yet a high reward. Most of the traditional behavioral interventions require in-person service delivery; however, this approach has several limitations from the implementor’s standpoint such as lack of consistent data collection, continuous monitoring, scalability, and sustainability of the intervention. AI chatbots have a low threshold for integration into these traditional services because they do not put a strain on existing resources such as experts, time, money, and effort. The chatbots can be freely deployed through daily use platforms and accessed at any time by the users. The use of chatbots can help integrate behavioral interventions into the daily clinical setting and avoid addition pressure faced by health care providers. For example, chatbots can independently offer low-intensity services such as information delivery to users. Furthermore, chatbots can offer provider-recommendation services, wherein, based on the analysis of real-time user data, the chatbots may offer suggestions to the health care providers to help them offer more effective services [27]. Therefore, public health professionals and health care providers can consider the integration of AI chatbots into existing services as a support tool, rather than a replacement [9].

Most of the studies (10/15, 67%) had a large and diverse sample population, demonstrating the potential for scaling up chatbot-based interventions. Almost half of the studies had >200 participants, with 27% (4/15) of studies consisting of a sample size ranging from approximately 920 to 991,217 participants. Similarly, the selected studies not only included samples with diverse health and behavioral conditions (13/15, 87%), such as breast cancer, smoking, obesity, unhealthy eating patterns, lack of physical exercise, conditions requiring medication, substance misuse, but also samples with no preexisting conditions (2/15, 13%). This demonstrates the potential of AI chatbots to reach a large and diverse population in different settings. This is because AI chatbots have the potential to be integrated into extensively used existing platforms such as text SMS, Facebook Messenger, and WhatsApp and deployed through commonly used devices such as smartphones, computers, and Alexa, making it highly feasible to access a large and diverse population. This finding is consistent with the previous systematic reviews that reported the integration of AI chatbots into diverse platforms, such as Slack (Slack Technologies, LLC), Messenger, WhatsApp, and Telegram [1,2,14], and the use of a large sample size, such as >100 participants in 10 of 15 studies [21,23,24,26,27,29-33]. Thus, public health professionals can deploy AI chatbots for education, the promotion of behavior change, and the provision of health care services to prevent health issues that affect a large population.

### Limitations of the Reviewed Studies and Future Research Directions

#### Nascent Application of AI Chatbots

Almost 75% (11/15) of the articles were published in the years 2019 and 2021, indicating that the use of AI-driven chatbot interventions for behavior changes is at a nascent stage. Most studies (9/15, 60%) adopted a pre-post study design with no control group, with only 27% (4/15) of studies using RCT models, reinstating the immaturity in establishing causal connections between AI-based conversational agents and health behavior outcomes. This finding is aligned with many previous systematic reviews that reported that 4 of 9 studies were RCTs, remaining were quasiexperimental, feasibility, or pilot RCT studies [9], 2 of 10 studies were RCTs, majority were quasiexperimental [14], and 2 of 17 studies were RCTs, majority were quasiexperimental [1]. Future studies need to adopt robust RCTs that can establish a causal relationship between AI chatbots and health outcomes.

#### Risk of Internal Validity

The outcome of this review should be interpreted with caution because of the moderate to high risk of internal validity within the selected studies. In the included studies, the risk of outcomes from unintended sources was high owing to the lack of information on the measures to avoid the influence of other interventions and level of adherence to the intervention protocol. The risk of bias in the measurement of the outcomes was moderate to high owing to the lack of concealment of the assigned intervention from the evaluators and the lack of using validated and reliable outcome measures. The risk of bias in the analysis was moderate to high owing to high dropout rates, the lack of power calculation to estimate sample size, and the lack of information on the use of intent-to-treat analysis. These findings are consistent with many previous systematic reviews that reported moderate risk of outcomes from unintended sources owing to confounding in all quasiexperimental studies [9]; high risk of outcome measurement because evaluators were aware of the assigned intervention [8,9] or nonvalidated instruments were used for outcome measurement [1,11]; and moderate risk of bias in analysis owing to high attrition rate, the lack of analysis methods for bias correction, the lack of power analysis, and small sample size at follow-up [2,9].

There was also inconsistency across studies in the measures of secondary outcomes, that is, feasibility, usability, acceptability, and engagement. This finding is consistent with most of the previous systematic reviews that reported mixed findings on secondary outcome measures [1,2,7,9-11]. First, this issue stems from the lack of a common operational definition for secondary outcomes in the context of chatbot-based interventions. Second, because the AI chatbot intervention domain is relatively new, there are very few measures on feasibility, usability, acceptability, and engagement with tested reliability and validity. Therefore, the researchers in the selected studies had to develop their own measures for assessing outcomes. This led to inconsistency in the measures and their operational definitions across the studies. Future studies should shape the development of common operational definitions for each of these outcomes to enable comparison and standardized reporting. Furthermore, future AI-chatbot–based intervention studies should follow the National Institutes of Health’s quality assessment criteria for controlled intervention studies [19] to assess their studies’ internal validity.

#### Lack of Description of AI Algorithm

In this review, most studies (14/15, 93%) did not describe the characteristics and handling of the input data, along with other processes related to the AI algorithm. This finding is consistent with the previous systematic literature review that reported inconsistent use of AI-software taxonomy and lack of depth of reported AI techniques and systems [14]. In alignment with CONSORT-AI extension [20], future studies need to elaborate on the following components related to the AI algorithm: (1) the process of supplying input data to the AI algorithm, including the user interface that enables data collection, inclusion or exclusion criteria of input data, handling of unavailable data, and establishing the credibility of the data collected (eg, specifying the source of input data); (2) the output by the AI algorithm and its relevance to the health-related goals; (3) the AI functioning, including the type of personalization algorithm such as ML, NLP, etc, version of the AI algorithm, and the accuracy level of the algorithm; (4) performance backlogs in the AI algorithm deployed, which would indicate the level of safety in using AI algorithms, especially with vulnerable populations; (5) the level and type of expertise required to integrate and successfully deploy the AI algorithm; and (6) the skills needed by the participants to use the AI chatbot, which would indicate the number of resources required and the feasibility of using AI algorithms

#### Lack of Generalizability

The selected studies were not representative of diverse geographies, cultures, and age groups, which exerted a strong bias on the generalizability of the studies. Out of the 13 studies that reported the geographical locations, all (100%) were conducted in the high-income countries; the majority of the studies (80%) were embedded in the Western culture, apart from the studies in South Korea and Japan; and most of the studies (>80%) were implemented with adults (≥18 years). These findings are consistent with the previous systematic literature reviews that reported that all the chatbot intervention studies were conducted in high-income countries [2,10,11,14], most studies were conducted with adults [2,7], and most studies did not focus on racial or ethnic minorities [2,14].

To increase the generalizability of the efficacy and feasibility of AI chatbots, future studies need to test their use in low-income countries or low-resource settings and with children and adolescents. The increased mobile connectivity and internet use in low-income countries [38] offer the potential to implement AI chatbot–based health behavior interventions. The use of AI chatbots can tackle the challenges faced by the health systems in low-income countries, such as the lack of experts, limited health infrastructure in rural areas, and poor health access [39]. Similarly, with the rise in the use of smartphones and latest digital technologies among adolescents [40], AI chatbots offer the opportunity to deliver engaging behavioral health interventions to them. The nonjudgmental and nonstigmatic attributes of AI chatbot–based interventions offer a solution to the challenges faced by adolescents in seeking behavioral health services, such as perceived and enacted stigma and lack of motivation [9,40].

#### Safety and Ethics

In this review, the evidence for patient safety was limited; however, the limited evidence stated that chatbots were safe for behavioral and mental health interventions. Only 7% (1/15) of studies, that is, the study by Maher et al [22], reported safety in terms of the absence of adverse events. This finding is consistent with the previous systematic literature reviews that reported very few studies discussed participant safety or ethics in terms of adverse events [1,2,7,9] and data security or privacy [2,8]. The occurrence of flexible, real-time, and large number of conversations with AI chatbots increases the probability of error by the AI algorithm. This can lead to unintended adverse outcomes, especially in the case of sensitive topics. Therefore, in the context of the nascent use of AI technologies, future studies should assess and report AI performance from ethical and safety standpoints.

### Limitations of This Review

This systematic literature review has several limitations. First, a meta-analysis was not conducted for the reviewed studies. Owing to heterogeneity in the research design, outcomes reported, and outcome measures, a meta-analysis was not perceived as feasible by the authors. Second, this review did not cover a comprehensive set of behavioral outcomes. The selected studies focused on only 3 behavioral outcomes: healthy lifestyle (physical activity and diet), smoking cessation, and treatment or medication adherence. However, this was also because the authors had adopted strict inclusion criteria for AI chatbots, and studies with rule-based chatbots were ruled out, restricting the number of behavioral outcomes covered. Third, the data matching for the tables was not quantified was not quantified; therefore, intercoder reliability was not reported. However, data extraction and quality assessment were conducted by 2 authors independently, followed by a discussion among the authors to finalize tables. Fourth, articles from outside selected databases (eg, Google Scholar), unpublished work and conference articles, gray literature (eg, government reports), and articles in other languages were not included. Fifth, intervention studies that did not provide a clear description of AI chatbots or did not label AI chatbots as a keyword were excluded.

### Conclusions

This review provides an evaluation of AI chatbots as a medium for behavior change interventions. On the basis of the outcomes of the selected studies (N=15), AI chatbots were efficacious in promoting healthy lifestyles (physical activity and diet), smoking cessation, and treatment or medication adherence. However, the studies had mixed results in terms of the feasibility, acceptability, and usability of AI chatbots in diverse settings with diverse populations. The efficacious outcomes of AI-driven chatbot interventions can be attributed to the fundamental characteristics of an AI chatbot: (1) personalized services, (2) nonjudgmental safe space to converse, (3) easy integration into existing services, (4) engaging experience, and (5) scalability to a large and diverse population. However, the outcomes of this review need to be interpreted with caution because most of the included studies had a moderate risk of internal validity, given that the AI chatbot intervention domain is at a nascent stage. Future studies need to adopt robust RCTs and provide detailed descriptions of AI-related processes. Overall, AI chatbots have immense potential to be integrated into existing behavior change services owing to their (1) the ease of integration; (2) potential for affordability, accessibility, scalability, and sustainability; (3) delivery of services to vulnerable populations on sensitive issues in a nonstigmatic and engaging manner; and (4) the potential for consistent data collection to support health care providers’ decisions.

## Appendix

Multimedia Appendix 1 Search string.

Multimedia Appendix 2 Methodology assessment based on the National Institutes of Health’s quality assessment tool for controlled intervention studies.

Multimedia Appendix 3 Quality assessment of chatbot interventions based on CONSORT-AI (Consolidated Standards of Reporting Trials–Artificial Intelligence extension).

Multimedia Appendix 4 Outcomes of the reviewed articles.

Multimedia Appendix 5 Features of the chatbots in the reviewed studies.

## Abbreviations

AI: artificial intelligence
CASC: CureApp Smoking Cessation
CBT: cognitive behavioral therapy
CONSORT-AI: Consolidated Standards of Reporting Trials–Artificial Intelligence
HCAI: Lark Health Coach
HLCC: Healthy Lifestyle Coaching Chatbot
ML: machine learning
NLP: natural language processing
PRISMA: Preferred Reporting Items for Systematic Reviews and Meta-Analyses
RCT: randomized control trial
SFA: Smoke Free app
TTM: transtheoretical model
