# Supplementary material for: Artificial Intelligence–Based Chatbots for Promoting Health Behavioral Changes: Systematic Review
Source: J Med Internet Res. 2023 Feb 24;25:e40789. doi: 10.2196/40789 (PMC10007007; doi:10.2196/40789)
Supplement: Multimedia Appendix 1 [file jmir_v25i1e40789_app1.docx]

This is a Multimedia Appendix to a full manuscript published in the J Med Internet Res. For full copyright and citation information see http://dx.doi.org/10.2196/jmir.40789

Appendix 1

PubMed Search Strategy

Chatbot OR chatterbot OR “chatter robot” OR “artificial intelligence” OR “conversational AI” OR “conversational agency” OR “virtual agent” OR “conversational agents” OR bot AND “health promotion” OR “health behaviors” OR “behavior change” OR “substance use” OR “alcohol use” OR drinking OR “cigarette use” OR smoking OR “drug abuse” OR “drug use disorder” OR “risk behaviors” OR lifestyle OR exercise OR “nutrition behavior” OR sleep OR adherence OR “body weight” OR “physical activity” OR diet OR “risky behaviors” OR “healthcare seeking behaviors” OR “prescribed medical treatment” OR “tobacco use” OR vaping AND intervention.

Same key words and logical operators were used for other databases.
