# Supplementary material for: Artificial Intelligence–Based Chatbots for Promoting Health Behavioral Changes: Systematic Review
Source: J Med Internet Res. 2023 Feb 24;25:e40789. doi: 10.2196/40789 (PMC10007007; doi:10.2196/40789)
Supplement: Multimedia Appendix 2 [file jmir_v25i1e40789_app2.docx]

This is a Multimedia Appendix to a full manuscript published in the J Med Internet Res. For full copyright and citation information see <http://dx.doi.org/10.2196/jmir.40789>.

**Appendix 2**. Methodology assessment based on the NIH quality assessment tool for controlled intervention studies.

|  | Study | | Prespecified outcomes | Described randomized | Treatment allocation | Blinding | Similarity of groups at baseline | Concealment of assigned intervention | Avoid other intervention | Adherence | Validity and reliability of outcome measures | Dropout | Power calculation | Intention-to-treat analysis | Total Score (%) |
| --- | --- | --- | --- | --- | --- | --- | --- | --- | --- | --- | --- | --- | --- | --- | --- |
| 1 | Piao et al [21] | | + | + | + | + | + | NR | NR | NR | + | - | + | NR | 7/12, 58.33% |
| 2 | Maher et al [22] | | + | NA | NA | NR | NA | NR | NR | NR | + | + | + | + | 5/9, 55.56% |
| 3 | Carrasco-Hernandez et al [23] | | + | + | + | + | + | NR | NR | NR | + | - | + | + | 8/12, 66.67% |
| 4 | Stephens et al [6] | | + | NA | NA | NR | NA | NR | NR | NR | ? | NR | NR | NR | 1/9, 11.11% |
| 5 | Perski et al [24] | | + | + | + | + | - | NR | NR | NR | ? | - | + | + | 6/12, 50% |
| 6 | Masaki et al [25] | | + | NA | NA | NR | NA | NR | NR | NR | + | + | NR | + | 4/9, 44.44% |
| 7 | Chaix et al [26] | | + | NA | NA | NR | NA | NR | NR | + | + | - | NR | NR | 3/9, 33.33% |
| 8 | Calvaresi et al [27] | | + | NA | NA | NR | NA | NR | NR | NR | NR | NR | NR | NR | 1/9, 11.11% |
| 9 | Galvão Gomes da Silva et al [5] | | + | NA | NA | NR | NA | NR | NR | NR | ? | NR | NR | NR | 1/9, 11.11% |
| 10 | Stein & Brooks [28] | | + | NA | NA | NR | NA | NR | NR | NR | NR | + | NR | NR | 2/9, 22.22% |
| 11 | Crutzen et al [29] | | + | NA | NA | NR | NA | NR | NR | NR | NR | + | NR | NR | 2/9, 22.22% |
| 12 | Brar Prayaga et al [30] | | + | NA | NA | NR | NA | NR | NR | + | + | + | NR | + | 5/9, 55.56% |
| 13 | Prochaska et al [31] | | + | NA | NA | NR | NA | NR | NR | NR | + | - | + | + | 4/9, 44.44% |
| 14 | To et al [32] | | + | NA | NA | NR | NA | NR | + | NR | + | + | NR | NR | 4/9, 44.44% |
| 15 | Bickmore et al [33] | | + | + | + | NR | + | NR | NR | NR | + | - | NR | NR | 5/12, 41.67% |
| + = | | 1, Compliant | | | | | | | | | | | | | |
| ? = | | 0, Not clear | | | | | | | | | | | | | |
| - = | | 0, Not compliant | | | | | | | | | | | | | |
| NR = | | 0, Not reported | | | | | | | | | | | | | |
| NA = | | Not applicable | | | | | | | | | | | | | |
