# Supplementary material for: Artificial Intelligence–Based Chatbots for Promoting Health Behavioral Changes: Systematic Review
Source: J Med Internet Res. 2023 Feb 24;25:e40789. doi: 10.2196/40789 (PMC10007007; doi:10.2196/40789)
Supplement: Multimedia Appendix 3 [file jmir_v25i1e40789_app3.docx]

This is a Multimedia Appendix to a full manuscript published in the J Med Internet Res. For full copyright and citation information see http://dx.doi.org/10.2196/jmir.40789

**Appendix 3.** Quality assessment on Chatbot interventions based on CONSORT guidance – AI extension

|  | Study | Statement of AI algorithm | Use of AI intervention in context of the clinical pathway | Inclusion and exclusion criteria at the level of the input data | Description of the approaches to handle unavailable input data | Describe input data acquisition process for AI intervention | Specifications of human-AI interaction in the collection of input data | Output of the AI algorithm | Explanations of how AI intervention’s outputs contribute to health behavior changes |  |
| --- | --- | --- | --- | --- | --- | --- | --- | --- | --- | --- |
| 1 | Piao et al [21] | + | + | NR | NR | + | + | + | + |  |
| 2 | Maher et al [22] | + | + | NR | NR | NR | NR | + | + |  |
| 3 | Carrasco-Hernandez et al [23] | + | + | NR | NR | + | NR | + | + |  |
| 4 | Stephens et al [6] | + | + | NR | NR | + | NR | + | + |  |
| 5 | Perski et al [24] | + | + | NR | NR | + | NR | + | + |  |
| 6 | Masaki et al [25] | + | + | NR | NR | + | + | + | + |  |
| 7 | Chaix et al [26] | + | + | NR | NR | + | + | + | + |  |
| 8 | Calvaresi et al [27] | + | + | NR | NR | + | + | + | + |  |
| 9 | Galvão Gomes da Silva et al [5] | + | + | NR | NR | + | + | + | + |  |
| 10 | Stein & Brooks [28] | + | + | NR | NR | + | + | + | + |  |
| 11 | Crutzen et al [29] | + | + | NR | NR | + | NR | NR | NR |  |
| 12 | Brar Prayaga et al [30] | + | + | + | + | + | + | + | + |  |
| 13 | Prochaska et al [31] | + | + | NR | NR | + | + | + | + |  |
| 14 | To et al [32] | + | + | NR | NR | + | + | + | + |  |
| 15 | Bickmore et al [33] | + | + | NR | NR | NR | NR | NR | NR |  |
| +  NR | Described  Not reported | | | | | | | | | |
