# Supplementary material for: Artificial Intelligence–Based Chatbots for Promoting Health Behavioral Changes: Systematic Review
Source: J Med Internet Res. 2023 Feb 24;25:e40789. doi: 10.2196/40789 (PMC10007007; doi:10.2196/40789)
Supplement: Multimedia Appendix 4 [file jmir_v25i1e40789_app4.docx]

This is a Multimedia Appendix to a full manuscript published in the J Med Internet Res. For full copyright and citation information see http://dx.doi.org/10.2196/jmir.40789

**Appendix 4.** Outcomes of reviewed articles

| Study | Feasibility | Acceptability | Usability | Health behavior outcomes (primary outcome) |
| --- | --- | --- | --- | --- |
| Piao et al [21] |  |  |  | (1) Habit strength (Self-Report Habit Index (SRHI)) (intervention vs. control)  Results:  - Significant between-group differences in SRHI when controlled for intrinsic award via app (*p*=.008)  - Non-significant between-group differences in SRHI, apart from physical activity (*p*=.045), when not controlled for intrinsic rewards via app (*p*=.21)  Analytical methods and results: Repeated measures analysis of variance (ANOVA) |
| Maher et al [22] | (1) *Safety*  Results: No adverse events were reported.  (2) *Retention rate*  Results: 5 withdrawals (83.9% retention rate)  (3) *Duration of engagement*  a) 64% check-ins completed on average by participants  b) Decrease in check-ins by 20% (mid program) followed by an increase in final weeks |  | (1) *Ease of Use*  Results: Participants had minimal smartphone skills  (2) *Content usability*  Results: Difficulty in consuming the recommended number of food group servings | (1) Physical activities (total minutes of weekly physical activity; Active Australia Survey) (week 12 vs baseline)  Results: *Mean* improvement 109.8 minutes [95% *CI* (1.9, 217.7)]  [*F*(2, 29) = 6.45, *p* = .005]  (2) Adherence to healthy (Mediterranean) diet (Australian Mediterranean diet adherence tool) (week 12 vs baseline)  Results: *Mean* improvement by 5.7 points [95% *CI* (4.2, 7.3)]  [*F*(2,29) = 44.45, *p* < .001]  (3) Weight (Kg)  (week 12 vs baseline)  Results: Decrease in the average weight by 1.3 Kgs [95% *CI* (–0.1, –2.5)]  [*F*(2, 29) = 5.41, *p* = .01]  (4) Waist circumference (cm)  (week 12 vs baseline)  Results: Decrease in the average waist circumference by 2.1 cm [95% *CI* (–3.5, –0.7)]  [*F*(2, 29) = 7.13, *p* = .003]  (5) Systolic and diastolic blood pressure (Omron Healthcare)  Results: Mean improvement 0.2 [*F*(2,29) = 0.11, *p* = .90], and 1.0 [*F*(2,29) = 0.64, *p* = .54], respectively.  Analytical methods: Repeated measures ANOVA |
| Carrasco-Hernandez et al [23] | (1) *Duration of engagement* (the ratio of rated messages divided by the total number of message)  Results: Engagement was the highest at first month and reduced gradually, becoming lowest at 12 months. A significant difference was found in system engagement at 6 months (*p*=.04) but not in all subsequent months. |  | (1) *Content usability* (Perceived quality questionnaire, range: 1 to 5)  Results: averagely high scores:  E.g., “I felt in control of modifying my interest profile” (*Mean* = 3.63, *SD* = 0.66); “The messages recommended to me were diverse (*Mean* = 4.47, *SD* = 0.51) | (1) Smoking abstinence rate (exhaled carbon monoxide and urine cotinine tests)  Results: *OR* = 2.15 [95% *CI* (1.13, 4.08), *p* = .02] (intervention vs. control; 12 months)  Analytical method: Multinomial model  (2) Health-related quality of life (Short-Form Health Survey and EuroQo1)  Results: *OR* = 0.17 [95% *CI* (-6.36, 6.70)] (intervention vs. control; at 6 months); *OR* = 3.01 [95% *CI* (-3.14, 9.16)] (at 12 months)  (3) Healthy lifestyle (body mass index)  Results: *OR* = -0.09 [95% *CI* (-0.77, 0.60), *p* = .80] (intervention vs. control; at 6 months); *OR* = 0.25 [95% *CI* (-0.53, 1.03), *p* = .52] (at 12 months)  (4) Physical activity (International Physical Activity Questionnaire)  Results: *p* = .47 (at 6 months); *p* = .73 (at 12 months) (intervention vs. control)  Analytical methods:  Multinomial model and chi-square test |
| Stephens et al [6] | (1) *Messages exchanged*  Results:  The total number of conversations with chatbot per month  Results: 4123 (*Mean* = 90 conversations per person)  (2) *Proportion of user-initiated conversation* (ratio of Chatbot-initiated vs. patient-initiated conversations)  Results: 26.4% were patient-initiated  (3) *Duration of engagement* (duration of conversations)  Results: average duration of conversation (*Mean* = 12.5 minutes, *SD* = 15.62) |  | (1) *Content usability* (self-reported endorsement of helpfulness during the conversation and percent times progressed toward the target goals):  Results: 96% and 81% of the time respectively.  (2) *Outside office support*  Results: Support provide outside office (sustainability)  Results: 17.8% of total support (55 hours and 45 minutes) |  |
| Perski et al [24] | (1) *Duration of engagement* (automatically recorded number of logins) (intervention vs. control)  Results: *Median* = 16, *IQR* = 65.5 (intervention) vs. *Median* = 5, *IQR* = 22 was associated with 107% increase in engagement (*p* < .001)  Analytical methods: Negative binomial regression analyses |  |  | (1) Smoking abstinence (continuous abstinence)  Results: Intervention group had 2.44 times greater odds of being abstinent. *AOR* = 1.36 [96% *CI* (1.16, 1.61), *p* < .001]  Analytical methods: logistic regression |
| Masaki et al [25] | (1) *Duration of engagement*  Results:  a) Number of days of diary entries (from weeks 0 to 12)  Results: *Mean* = 56.1 (*SD* = 31.3)  b) Number of educational videos viewed from start to finish  Results: *Mean* = 12.6 (*SD* = 6.8) | (1) *Satisfaction*  Results:  a) Number of times "Like!" tapped to the advice provided by the chatbot (from week 0 to 12)  Results: *Mean* = 26.5 times (*SD* = 63.8) | (1) *Content usabilit*y  Results:  a) Number of times "Like!" tapped to the advice provided by the chatbot (from week 0 to 12)  Results: *Mean* = 26.5 times (*SD* = 63.8)  b) Number of times “Call” tapped to for AI nurse in context of smoking impulses or side effects (from weeks 0 to 12)  Results: *Mean* = 1.7 times (*SD* = 2.4) | (1) Smoking abstinence (continuous abstinence rate)  Results: 76% [95% *CI* (65, 88)] (12 weeks), 64% [95% *CI* (51, 76)] (24 weeks), 58% [95% *CI* (46, 71] (52 weeks)  (2) Cigarette withdrawal (Mood and Physical Symptoms scale)  Results: Differences = -6.4 (*SD* = 5.8) (12 weeks – baseline)  (3) Tobacco craving (12-item French version of the Tobacco Craving Questionnaire)  Results: Differences = -0.6 (*SD* = 1.5) (12 weeks – baseline)  (4) Social nicotine dependence (Kano Test for Social Nicotine Dependence)  Results: Differences = -6.7 (*SD* = 5.2) (12 weeks – baseline) |
| Chaix et al [26] | (1) *Messages exchanged*  The total number of conversations with chatbot per month  Results: 132970 (*Mean* = 139 conversations per person)  (2) *Retention rate* (users who send at least one message per month)  Results: 72% (2nd month) reduced to 31% (8^th^ month) | (1) *Satisfaction*  Results:  a) Rate of participants who reported satisfaction with the use of chatbot  Results: 93.95% (900/958)  (2) *Non-judgmental safe space*  Results: Share personal and intimate information such as sexuality. | (1) *Content usability*  Results:  a) Rate of participants who reported satisfaction with the use of chatbot  Results: 93.95% (900/958)  b) Rate of participants who reported that chatbot was supportive or helpful to track their treatment effectively  Results: 88% (943/958) | (1) Medication adherence rate (The average compliance of patients using the prescription reminder feature)  Results: More than 20% improvement in 4 weeks (*p* = .04)  Analytical methods: *t*-test |
| Calvaresi et al [27] | (1) *Duration of engagement* (rate of participants operating the app on social network)  Results: 74% (200/270) users engaged actively |  |  | (1) Rate of participants who succeeded in the smoking cessation goal, three months after the last cigarette  Results: 28.9% (78/270) participants succeeded in the smoking cessation goal  (2) Rate of the improvement on obtained cessations compared to previous campaign without a chatbot  Results: 10% |
| Galvão Gomes da Silva et al [5] |  | (1) *Non-judgmental safe space*  Results: Chatbot offered space and time to talk | (1) *Easy of use*  Results: Easy to tap on the robot’s head to continue the conversation.  (2) *Content usability*  Results: Clear and easy to understand interview script, but with some ambiguity. | Qualitative data  (1) Interview evaluation  - Connection: Tension or awkwardness, followed by comfortable experience.  (2) Motivation  - Enhanced immediate motivation  (3) Engagement of physical activity after the program  - Mixed reports  (4) Positive features  - Flexible and increased self-awareness and social skills. |
| Stein & Brooks [28] | (1) *Duration of engagement* (time in weeks between first and final use of the app)  Results: The average duration of app use was 15 weeks (*SD* 1.0), and users averaged 103 sessions each. |  | (1) *Content usability*  Results:  a) In-app user trust survey  Results: 100% response rate and positive results  b) Average satisfaction score  Results: 87 out of 100  c) Average net promoter score (promoters (9-10) – detractors (0-6))  Results: 47  d) Average scores for disappointment if app not offered  Results: 6.73 | (1) Weight loss (percent weight change)  Results: Weight loss (standard error of the mean) was 2.38% (0.69%) of baseline weight in  75.7% (53/70) of users.  (2) Meal quality (if they contained healthy food and no unhealthy food)  Results: Percentage of healthy meals increased by 31%, and the percentage of unhealthy meals decreased by 54%. |
| Crutzen et al [29] | (1) *Messages exchanged*  Results: The total number of conversations with chatbot per month  Results: 42217 (*Mean*= 11.3 conversations per person)  (2) *Duration of engagement*  Results:  a) The average duration of chatbot use per person = 45 min  b) The average duration of a conversation per person  Results: *Mean* = 3 minutes 57 seconds | (1) *Non-judgmental safe space*  Results: Chatbot was used for questions regarding sex, drugs, and alcohol, and was considered more anonymous and faster than information lines | (1) *Ease of use* (visual analog scale)  Results: Low (N = 852, M =47.8, *SD* =31.4)  (2) *Content usability*  Results:  a) Reliability of the information provided by chatbot  Results: High (N = 852, M =56.4, *SD* =51.5)  b) Chatbot was considered easier to use as compared with information lines  c) Chatbot was considered to provide better quality and concise of information in comparison with search engines.  d) The quantity of information of the chatbot was considered less in comparison with both information lines and search engines |  |
| Brar Prayaga et al [30] |  |  |  | (1) Refill requests from participants (rate = requests/total reminders)  Results: 17.40% (47,552/273,356)  Analytic methods: chi-square test (*χ^2^*) |
| Prochaska et al [31] | (1) *Messages exchanged*  Results: 600.7 (SD 556.5) sent messages.  (2) *Duration of engagement*  Results:  (a) Participants’ W-SUDs use averaged 15.7 (*SD* 14.2) days, 12.1 (*SD* 8.3) modules.  (b) About 94% (562/598) of all completed psychoeducational lessons were rated positively. | (1) *Acceptability*  (a) Most participants reported receiving the service they desired (41/51, 80%) and would recommend W-SUDs to a friend (39/51, 76%). | (1) *Content usability*  Results:  (a) Fewer felt Woebot met most or all of their needs (22/51, 43%). | (1) Significant increase in confidence to resist urges to use substances (mean score change +16.9, *SD* 21.4; *p*<.001)  (2) Significant decrease in substance use occasions (mean change -9.3, SD 14.1; *p*<.001), Alcohol Use Disorders Identification Test-Concise (mean change -1.3, SD 2.6; *p*<.001), 10-item Drug Abuse Screening Test (mean change -1.2, SD 2.0; *p*<.001), Patient Health Questionnaire-8 item (mean change 2.1, SD 5.2; *p*=.005), Generalized Anxiety Disorder-7 (mean change 2.3, SD 4.7; *p*=.001), and cravings scale (68.6% vs 47.1% moderate to extreme; *p*=.01). |
| To et al [32] | (1) *Message frequency*  (a) On average, 6.7 (SD 7.0) messages/week were sent to the chatbot.  (b) About half of the participants (58/113) sent messages to the chatbot at least once a day.  (2) *Duration of engagement*  (a) Participants spent 5.1 minutes with the chatbot per day.  (b) 71/113, 62.8% participants always read the chatbot messages. | (1) *Satisfaction*  (a) About one-quarter liked very much the messages that the chatbot sent out (26/113, 23%).  (b) Only 43.4% (49/113) thought that the chatbot understood their messages most of the time.  (c) About one-third would continue to use the chatbot in the future (40/113, 35.4%). | (1) *Content usability*:  Results:  (a) Most of the participants scored overall the usability of the chatbot (101/113, 89.4%) as at least “OK” (System Usability Scale).  (b) Average usability score for the chatbot was (49/113, 43.4%), including increase in confidence (52/113, 46%), overcoming barriers (60/113, 53.1%), increased support (59/113, 52.2%), planning (63/113, 55.7%), staying motivated (47/113, 41.6%), and becoming more active (60/113, 53.1%).  (2) *Technical issues*  Most participants experienced technical issues (93/113, 82.3%) and stopped receiving the chatbot messages at any time during the study (95/113, 84.1%). | At follow-up, results:  (1) Participants recorded more steps (increase of 627, *p*<.01) and,  (2) More total physical activity (increase of 154.2 min/week; 3.58 times higher at follow-up, *p*<.001).  (3) The decrease in BMI was not significant (-0.13 *CI* -0.37 to 0.11)  (4) Participants were also more likely to meet the physical activity guidelines (*OR* 6.37, 95% *CI* 3.31-12.27) at follow-up. |
| Bickmore et al [33] | - | (1) *Satisfaction*  (a) Participants were above average satisfied with agent *Mean* = 4.30 (1.84) on a scale of 1 to 7.  (b) Participants reported below average desire to continue with agent *Mean* =3.75 (2.18) on a scale of 1 to 7. | (1) *Ease of use*  (a) Participants reported relative ease of using the chatbot *Mean* = 4.80 (1.97) on a scale of 1 to 7. | (1) No significant differences among conditions in International Physical Activity Questionnaire (IPAQ) (*F*(3,107)=1.07, *p*=0.367).  (2) A significant difference between conditions with the DIET intervention doing the best (*F*(3,103)=4.52, *p*=0.005).  (3) No significant differences among conditions for weight (*F*(3,105)=1.09, *p*=0.374). |
