# Supplementary material for: Artificial Intelligence–Based Chatbots for Promoting Health Behavioral Changes: Systematic Review
Source: J Med Internet Res. 2023 Feb 24;25:e40789. doi: 10.2196/40789 (PMC10007007; doi:10.2196/40789)
Supplement: Multimedia Appendix 5 [file jmir_v25i1e40789_app5.docx]

This is a Multimedia Appendix to a full manuscript published in the J Med Internet Res. For full copyright and citation information see http://dx.doi.org/10.2196/jmir.40789

**Appendix 5.** Chatbot features of reviewed studies

| Study | Chatbot Name | Chatbot types | Chatbot components/functionality | Settings (devices, duration, dose) | Existing AI-technology | Input data sources for AI-algorithm | Platform | Theoretical foundation | AI algorithm |  |
| --- | --- | --- | --- | --- | --- | --- | --- | --- | --- | --- |
| Piao et al [21] | Healthy Lifestyle Coaching Chatbot (HLCC) | Text-based | (1) *Behavioral goals setting:* Set behavioral goals and provide reminders (set push alarms on daily routine with basic conditions: who, when, where, what, and how much).  (2) *Behavioral monitoring:* monitor behavioral performance through data on performance content and pictures.  (3) *Motivation Reinforcement:* provide feedback based on the performed behaviors (extrinsic and intrinsic rewards). | Devices: Smartphone  Duration: 12 weeks  Dose: Daily | Watson Conversational tool | (1) User’s goals  (2) Feedback on behavioral performance | South Korean messenger app “KakaoTalk” | Habit formation model | N/A |  |
| Maher et al [22] | Paola | Text-based | (1) *Behavior-related information:* Educational sessions on physical activity, goal setting, self-monitoring, and Mediterranean diet.  (2) *Behavioral goal setting:* Set behavioral goals (physical activities and dietary).  (3) *Behavioral monitoring:* Weekly check-ins to monitor daily dietary performance and physical exercise.  (4) *Availability*: Available 24*7 to answer users’ questions. | Devices: Smartphone  Duration: 12 weeks  Dose: Daily | Watson Conversational tool | (1) Baseline characteristics  (2) User’s goals  (3) Feedback on behavioral performance | - | N/A | Natural language processing |  |
| Carrasco-Hernandez et al [23] | DigiQuit | Text-based | (1) *Behavior-related information:* Recommendations, and benefits of being a nonsmoker.  (2) *Behavioral monitoring:* Collection and presentation of physical activity data.  (3) *Motivation reinforcement:* Provide personalized motivational messages related to smoking abstinence according to TTM. | Devices: Smartphone  Duration: 12 months  Dose: Daily | - | (1) Data on chatbots’ usability | - | Transtheoretical model of behavioral change (TTM) | Hybrid Health Recommender System |  |
| Stephens et al [6] | Tess | (1) Text-based  (2) Voice-based | (1) *Emotional support:* Empathetic health counseling or compassionate care through ML-driven emotional algorithms.  (2) *Availability*: 24*7 continuous and unlimited AI-driven conversations at any time of the day. | Device: Smartphone  Duration: 10-12 weeks  Dose: N/A | - | (1) Clinical scripts targeted at behavior change  (2) Electronic Health Records (HER)  (3) Chatbots’ usability | (1) Facebook Messenger  (2) WhatsApp  (3) Google Home  (4) Amazon Alexa  (5) Mobile SMS | (1) Cognitive behavioral therapy  (2) Emotionally focused therapy  (3) Motivational interviewing | Machine-learning |  |
| Perski et al [24] | Smoke Free App (SFA) | (1) Text-based | (1) *Behavior-related information*: e.g., provide normative information about other’s experiences, explain the implications of abrupt cessation.  (2) *Behavioral goal setting:* Facilitate goal setting, prompt review of goals.  (3) *Motivation reinforcement:* Cost calculator and scoreboards used to reinforce milestones, cravings resisted, smoke free days, and other motivational messages to remain smoke free  (6) *Behavioral monitoring*: Virtual diary, graph, health indicators used to present current and past smoking behavior  (8) *Availability*: On demand support as and when needed | Device: Smartphone  Duration: 1 month  Dose: Daily | - | (1) Baseline characteristics  user’s goals | N/A | (1) Mohr’s Model of Supportive Accountability  (2) 44-item taxonomy of behavior change techniques | N/A |  |
| Masaki et al [25] | CureApp Smoking Cessation (CASC) | (1) Text-based | (1) *Behavior-related information*: provide personalized advice on how deal with symptoms  (2) *Motivation reinforcement:* Encouraging messages for smoking cessation at appropriate times.  (3) *Availability*: On demand emergency support via AI nurse.  (4) *Provide-recommender system*: Recommendations to physicians to offer appropriate advice and counseling support. | Device: Smartphone  Duration: 24 weeks  Dose: Daily | - | (1) National guidelines on counseling support  (2) Baseline characteristics | N/A | N/A | N/A |  |
| Chaix et al [26] | Vik | (1) Text-based  (2) Voice based | (1)  *Behavior-related information*: Provide medical information on breast cancer epidemiology, treatment, side effects, sport, fertility, sexuality, and diet.  (2)  *Behavioral monitoring:* Provide reminder to take medication. | Device: Smartphone and computer  Duration: 12 months  Dose: Daily | - | (1) Feedback on behavioral performance  (2) Chatbots’ usability | Messenger (Facebook) | N/A | Machine learning |  |
| Calvaresi et al [27] | SMAG | (1) Text-based | (1) *Motivation reinforcement*: provide motivation interactions, e.g., encouraging messages.  (2) *Behavioral monitoring*: tracking the state of participants.  (3) *Behavior-related information*: disseminate information, provide alternatives to smoking.  (4) *Availability*: Anytime on-demand support and support during periods of high cravings. | Device: Smartphone and computer  Duration: 16 weeks  Dose: Daily | - | (1) Baseline characteristics  (2) Feedback on behavioral performance | Messenger (Facebook) | N/A | N/A |  |
| Galvão Gomes da Silva et al [5] | NAO | (1) 3D robot | (1) *Motivation reinforcement:* Advantages and disadvantages of the status quo, optimism about change, intention to change, evocation of idea about change, hypothetical changes.  (2) *Emotional support*: Express empathy through humanized robot interaction.  (3) *Behavioral goal setting:* setting goal, arriving at a plan. | Device: Social robot  Duration: 1 week  Doze: Once | - | - | - | (1) Motivational Interviewing | Face tracking technology |  |
| Stein & Brooks [28] | Lark Health Coach (HCAI) | (1) Text-based | (1) *Emotional support:* mimicking health professionals’ empathetic health counseling.  (2) *Behavioral goal setting*: goal-acting and action planning module.  (3) *Behavioral monitoring*: self-monitoring module, tracking weight, meals, and snack weekly.  (4) *Availability*: Continuous and unlimited availability of app with anytime and immediate feedback. | Device: Smartphone  Duration: 7 months  Doze: Daily | - | (1) Diabetes program prevention curriculum (DPP)  feedback on behavioral performance | - | (1) Cognitive behavioral therapy | Machine Learning |  |
| Crutzen et al [29] | Chatbot Bzz | (1) Text-based | (1) *Behavior-related information*: topic related content on sex, drugs, and alcohol.  (2) *Availability*: 24*7 with real time feedback. | Device: Smartphone and computer  Duration: 11 months  Doze: N/A | - | - | - | - | - |  |
| Brar Prayaga et al [30] | mPulse Mobile | (1) Text-based | (1) *Behavioral monitoring*: provide weekly reminder test to patients due for refill | Device: Smartphone  Duration: 2 years  Dose: Daily | - | - | SMS text messaging | - | Hybrid technique of NLP and conversational AI or ML |  |
| Prochaska et al [31] | Woebot | (1) Text-based | (1) *Emotional support:* empathic responses with tailoring to users’ stated mood(s).  (2) *Behavioral goal setting*  (3) *Behavioral monitoring*: mood tracking and regular check-ins for maintaining accountability.  (4) *Motivation reinforcement*: a focus on motivation and engagement through individualized weekly reports to foster reflection.  (5) *Activities*: related to psychoeducation and psychotherapeutic skills. Tailored  conversations depending on user input and choice with mindfulness exercises, gratitude journaling, and/or reflecting upon patterns and lessons already covered. | Device: Smartphone  Duration: 2 months  Dose: Daily | - | - | - | (1) Dialectical behavior therapy (2) Motivational interviewing  (3) Mindfulness  (4) Cognitive Behavioral Therapy | Natural language processing algorithms |  |
| To et al [32] | Ida | (1) Text-based | (1) *Motivation reinforcement*:  sent out daily motivational messages in relation to goal achievement, and automatically adjusted the daily goals based on physical activity levels in the last 7 days.  (2) *Behavioral goal setting*: Automatically adjusting the daily activity goals based on the average physical activity level achieved during the 7 previous days.  (3) *Behavior-related information*: When requested by the participants, the chatbot also provided sources of information on the benefits of physical activity.  (4) *Behavioral monitoring*:  Checked participants’ activity history (i.e., the step counts/min that were achieved on any day). | Device: Fitbit Flex 1 (Fitbit LLC)  Duration: 6 weeks  Dose: Daily | Dialogflow (Google Inc), an advanced Google machine learning platform for creating conversational AI-chatbots. | - | Messenger (Facebook) | (1) COM-B model, the core of the Behavior Change Wheel, a behavioral system focusing on 3 components: capability, opportunity, and motivation. | Natural language processing |  |
| Bickmore et al [33] | Chat1** | (1) Text-based | (1) *Therapeutic assistance*: Therapeutic Dialog Actions (talk therapy).  (2) *Activities*: “Homework Actions” (homework assignments the user is asked to do outside of the agent counseling sessions).  (3) *Behavioral monitoring*:  Action stage of change may consist of reviewing progress since the last conversation, and conducting a problem solving dialog about specific barriers to change.  (4) *Behavior goal setting*:  Setting goals as a new homework assignment. | Device: Computers  Duration: 2 months  Dose: Daily | - | - | - | (1) Transtheoretical model  (2) Motivational interviewing  (3) Social cognitive theory | Procedural knowledge and epistemological knowledge |  |
| **Chat1: The chatbot name for Bickmore et al. [30] i.e., Chat1 was given by authors for ease of reporting. | | | | | | | | | | |
